# Risk factors of postoperative pulmonary complications in patients with asthma and COPD

**DOI:** 10.1186/s12890-017-0570-8

**Published:** 2018-01-09

**Authors:** Takanori Numata, Katsutoshi Nakayama, Satoko Fujii, Yoko Yumino, Nayuta Saito, Masahiro Yoshida, Yusuke Kurita, Kenji Kobayashi, Saburo Ito, Hirofumi Utsumi, Haruhiko Yanagisawa, Mitsuo Hashimoto, Hiroshi Wakui, Shunsuke Minagawa, Takeo Ishikawa, Hiromichi Hara, Jun Araya, Yumi Kaneko, Kazuyoshi Kuwano

**Affiliations:** Division of Respiratory Diseases, Department of Internal Medicine, Jikei University School of Medicine, 3-25-8, Nishi-Shimbashi, Minato-ku, Tokyo, Japan

**Keywords:** Risk factors, Postoperative pulmonary complication, Asthma, COPD, Eosinophils

## Abstract

**Background:**

Postoperative pulmonary complications (PPC) in patients with pulmonary diseases remain to be resolved clinical issue. However, most evidence regarding PPC has been established more than 10 years ago. Therefore, it is necessary to evaluate perioperative management using new inhalant drugs in patients with obstructive pulmonary diseases.

**Methods:**

April 2014 through March 2015, 346 adult patients with pulmonary diseases (257 asthma, 89 chronic obstructive pulmonary disease (COPD)) underwent non-pulmonary surgery except cataract surgery in our university hospital. To analyze the risk factors for PPC, we retrospectively evaluated physiological backgrounds, surgical factors and perioperative specific treatment for asthma and COPD.

**Results:**

Finally, 29 patients with pulmonary diseases (22 asthma, 7 COPD) had PPC. In patients with asthma, smoking index (≥ 20 pack-years), peripheral blood eosinophil count (≥ 200/mm^3^) and severity (Global INitiative for Asthma(GINA) STEP ≥ 3) were significantly associated with PPC in the multivariate logistic regression analysis [odds ratio (95% confidence interval) = 5.4(1.4–20.8), 0.31 (0.11–0.84) and 3.2 (1.04–9.9), respectively]. In patients with COPD, age, introducing treatment for COPD, upper abdominal surgery and operation time (≥ 5 h) were significantly associated with PPC [1.18 (1.00–1.40), 0.09 (0.01–0.81), 21.2 (1.3–349) and 9.5 (1.2–77.4), respectively].

**Conclusions:**

History of smoking or severe asthma is a risk factor of PPC in patients with asthma, and age, upper abdominal surgery, or long operation time is a risk factor of PPC in patients with COPD. Adequate inhaled corticosteroids treatment in patients with eosinophilic asthma and introducing treatment for COPD in patients with COPD could reduce PPCs.

## Background

Postoperative pulmonary complications (PPCs) and their management are clinically important in patients with asthma and chronic obstructive pulmonary disease (COPD). PPCs include bronchospasm, atelectasis, pulmonary infection, respiratory failure and exacerbation of chronic pulmonary disease [1]. The prevalence of PPCs in all surgeries was reported to be 2 to 19% [1, 2]. Because PPCs contribute to mortality and longer stays in the hospital [3], it is necessary to appropriately evaluate relevant risk factors and establish perioperative management strategies. There are two types of risk factors for PPCs. One is patient-related risk factors, such as aging and pulmonary function, and the other is procedure-related risk factors, such as anesthetic techniques and operating time [1].

Although Global INitiative for Asthma (GINA) [4] and Global initiative for chronic Obstructive Lung Diseases (GOLD) [5] documents describe the perioperative management, most of the evidence presented in these documents was reported from 1990 to 2000. More recent progress in treatments for pulmonary diseases, especially new inhalants and devices, has significantly improved patient quality of life. It has been reported that some video-associated surgeries could decrease the prevalence of postoperative complications [6, 7].

To reduce PPC, we implemented the preoperative pulmonary management program in our hospital over 10 years ago. In our management program, all surgical patients with an abnormal pulmonary function test undergo a medical examination performed by pulmonologists and are administered medical treatment and evaluated for risk factors for PPC development. However, most evidence regarding the risk factors for PPC was established more than 10 years ago, and recent advances in inhalant drugs have changed the clinical course in affected patients, especially those with asthma and COPD. It is therefore critically important to precisely evaluate the risk factors for PPC development during perioperative management and to use recently available inhalant drugs in patients with both asthma and COPD.

## Methods

### Definition of asthma

All patients with asthma were diagnosed by physicians or self-reported, when the patients had past medical histories of asthma, typical symptoms (cough, wheezing, circadian variation and seasonal episodes) and pulmonary function compatible with asthma. Severity at baseline and during exacerbation was defined according to the GINA document [4]. Recently, a category called asthma-COPD overlap (ACO) was proposed. However, the definition of ACO has not yet been confirmed. Furthermore, patients with asthma, including those potentially in the ACO category, are at high risk of intraoperative asthma attacks, and they therefore receive inhaled or systemic corticosteroid therapy to prevent bronchospasm as a serious PPC of asthma. Therefore, in the present study, we classified asthma patients with any smoking history, including ACO, as the asthma group.

### Definition of COPD

All patients were diagnosed with COPD by the physicians, based on a smoking history (smoking index >10 pack-years), post-bronchodilator forced expiratory volume in one second [(FEV_1_)/ forced volume capacity(FVC)] < 0.7 and the exclusion of other diseases [5].

### Study subjects

**Fig. 1:**
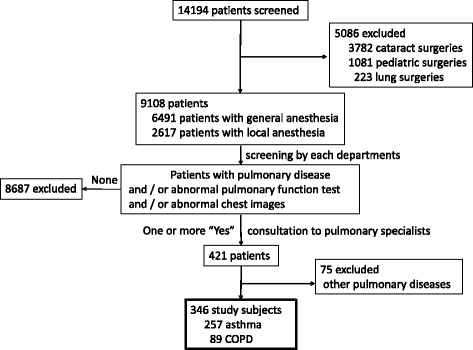
Study flow

Between April 2014 and March 2015, a total of 14,194 surgeries were performed in Jikei University hospital (Tokyo, Japan). We excluded 3782 cases of cataract surgery, 1081 pediatric surgeries (under 15 years old) and 223 cases of lung resection surgery from these cases. We excluded cataract surgery because of very short operation time, and also, we excluded lung resection surgery, because of a crucial risk factor for PPC by itself. The criteria for preoperative consultation by pulmonologists were as follows: (1) patients with any past or present pulmonary diseases, (2) patients with an abnormal pulmonary function test (spirometry) prior to general anesthesia, or (3) patients with abnormal chest images. After preoperative examinations, 421 patients met one or more of these criteria. The participants in the present study included 346 adult patients who consulted pulmonologists for asthma or COPD (Fig. 1). Patients with other pulmonary diseases were excluded from this study. Therefore, the operations covered in this study included those performed in the head, neck, limbs, abdomen, pelvis and cardiovascular system. This study was approved by the Ethical Committee of Jikei University (28–260(8503)).

### Data collection

We retrospectively examined the gender, age, underlying pulmonary diseases, medication, smoking index, peripheral blood eosinophil counts, pulmonary function test (volume capacity(VC), forced expiratory volume in one second (FEV_1_), FEV_1_/FVC, and %FEV_1_), Asthma Control Test (ACT), surgery-related factors (anesthetic technique, American Society of Anesthesiologists (ASA) physical status (PS), duration of operation time, and surgery site) and PPCs of all included patients. We defined PPC as bronchospasm, pneumonia, atelectasis, respiratory failure with oxygen therapy, and exacerbation of chronic pulmonary diseases within the first seven postoperative days [1, 8, 9]. We defined bronchospasm as symptoms (wheezing, shortness of breath, and cough) requiring additional medications, and we defined respiratory failure as hypoxia requiring more than oxygen therapy or prolonged oxygen therapy. We reviewed all medical records to measure these outcomes.

### Intervention

Patients consulted a pulmonologist four weeks before surgery. All smokers among the included patients were instructed to quit smoking. Our standard baseline therapy for bronchial asthma is based on the GINA stepwise approach [4]. We primarily targeted safe surgeries and referred to this document. To prevent perioperative bronchospasm, we increased the dose of inhaled corticosteroids(ICS) administered to most patients. After the first visit to our department, patients with severe/uncontrolled asthma revisited the department for a final decision regarding whether surgery was advisable. In patients with GINA STEP ≥ 3, neurosurgery, emergency surgery, PPC history, or low ACT scores (approximately <22) after ICS dose escalation, we introduced systemic corticosteroids, such as prednisolone (e.g., 20 mg/day, p.o.) for several days before surgery and a short-acting corticosteroid, such as hydrocortisone (e.g., 100 mg i.v. every 8 h) on the day of the surgery [10]. We recommended postponing the scheduled surgery in patients who had asthma exacerbation within two months. In patients with COPD, we introduced a long-acting muscarinic antagonist (LAMA), a long-acting beta 2 agonist (LABA) and a combination of ICS/LABA in accordance with the GOLD document [5]. Furthermore, in patients with very low pulmonary function, we recommended local anesthesia when possible.

### Statistical analysis

All statistical analyses were performed using StatView version 5 (SAS Institute Inc., Cary, NC, USA). All values are expressed as the mean ± standard deviation (SD). A *p*-value of <0.05 was considered statistically significant. The factors associated with patient characteristics were examined using a Mann-Whitney *U* test, Student’s *t* test, Fisher’s exact test, or chi-squared test (univariate model). Then, a logistic regression analysis was performed to evaluate the identified PPC risk factors (multivariate model), including age, gender (male), and other variables that achieved *p* < 0.20 in the univariate models. In patients with asthma, we selected age, gender (male), smoking index (≥20), peripheral blood eosinophil count (≥200/mm^3^), GINA STEP (≥3), %FEV_1_, upper abdominal surgery, perioperative systemic steroid therapy and operation time (minutes) for the multivariate analysis. On the other hand, in patients with COPD, we selected age, gender (male), COPD treatment, upper abdominal surgery and operation time (≥5 h) for the multivariate analysis.

## Results

Of the 421 patients who consulted pulmonologists for perioperative management, 257 had asthma and 89 had COPD. These patients included 186 males and 160 females. The mean age was 58.1 years old (range, 20–89 years old). The remaining 75 patients who met the consultation criteria had conditions including interstitial pneumonia, bronchiectasis, pulmonary infection, and malignant diseases.

We recommended changing the anesthetic to local anesthesia in 5 patients, because these patients had exacerbated chronic respiratory diseases or very low pulmonary functions. Furthermore, we recommended cancelling 19 surgeries, which were excluded from the analysis.

### Medical departments/services

A list of medical departments or services that performed surgical operations is provided in Table 1. Otolaryngology surgeries were the most frequent (177 patients) and 146 (82%) of these patients suffered from bronchial asthma.

**Table 1:** Medical departments that underwent surgical operations (*n* = 346)

Department	male	female	total
Otolaryngologic surgery	98	79	177 (51)
Orthopedic surgery	19	18	37 (11)
Gastrointestinal surgery	23	10	33 (9.5)
Urological surgery	22	7	29 (8.4)
Gynecological surgery	0	27	27 (7.8)
Hepatic surgery	7	4	11 (3.2)
Vascular surgery	6	3	9 (2.6)
Neurosurgery	4	5	9 (2.6)
Plastic surgery	5	3	8 (2.3)
Dermatological surgery	1	2	3 (0.9)
Breast surgery	0	2	2 (0.6)
Cardiac surgery	1	0	1 (0.3)

Data are presented as *n*, *n* (%)

### Patients’ characteristics associated with asthma and COPD

Patient characteristics associated with asthma and COPD are shown in Table 2. The mean age of the patients was younger in those with asthma than those with COPD. The mean FEV_1_/FVC was higher in the patients with asthma was than in the patients with COPD. In patients with asthma, disease severity at baseline was significantly associated with perioperative systemic corticosteroids treatment, and patients with severer asthma were treated with systemic corticosteroids (*p* < 0.0001, chi-square test, data not shown). A symptom questionnaire was performed at enrolment and completed by 77% of patients with asthma. The results revealed that there was a significant difference in ACT scores according to the GINA STEP (p < 0.0001, chi-square test, Table 3). Furthermore, there was a significant difference in ACT scores between the groups with and without perioperative corticosteroid treatment (22.3 ± 3.5 (*n* = 106) and 23.2 ± 2.2 (*n* = 92), respectively, *p* = 0.023, Student’s *t* test, data not shown).

**Table 2:** Clinical characteristics in asthma and COPD patients

	asthma (*n* = 257)	COPD (*n* = 89)
gender, male / female	111 / 146	75 / 14
age, years(range)	53.8 ± 16.0 (20–88)	70.6 ± 8.3 (49–89)
BMI, kg/m^2^	23.3 ± 4.5	23.0 ± 23.0
Smoking index, pack-years	9.4 ± 16.7	47.7 ± 33.6
(never/former/current)	(130 / 104 / 23)	(3 / 61 / 25)
%VC, %	110.5 ± 18.2	104.1 ± 20.5
FEV_1_/FVC, %	70.5 ± 12.2	59.3 ± 7.7
%FEV_1_, %	91.7 ± 19.6	84.5 ± 21.1
GINA STEP, 1/2/3/4/5	67 / 58 / 94 / 34 / 4	NA
CS (+), n_1_/n_2_/n_3_/n_4_/n_5_	25 / 19 / 60 / 25 / 4	NA
GOLD stage, 1/2/3/4	NA	47 / 35 / 6 / 1

Data are presented as n or mean ± SD (range)

*NA* not available

*CS* systemic corticosteroids

“nx” means that the number of patients whose GINA STEP is “x”

**Table 3:** ACT scores and GINA STEP

GINA STEP (n = 257)	1 (*n* = 67)	2 (*n* = 58)	3 (*n* = 94)	4 (n = 34)	5 (n = 4)	*p* value
ACT score (*n* = 198)	24.5 ± 1.0 (*n* = 45)	23.3 ± 1.9 (*n* = 43)	22.0 ± 3.0 (*n* = 81)	21.2 ± 4.5 (n = 25)	20.3 ± 5.5 (n = 4)	<0.0001*

Data are presented as n or mean ± SD

*chi-square test

### Postoperative pulmonary complications

In our study, 29 of 346 patients with asthma or COPD had PPCs. The details related to the PPCs and the clinical characteristics of the patients with asthma and COPD are shown in Tables 4, 5 and 6, respectively. Of the 257 patients with asthma, 18 (7.0%) had the PPC mild/moderate bronchospasm, and their symptoms promptly improved with additional treatment. There was a significant difference in ACT scores between the groups with and without PPCs (21.2 ± 4.8 (*n* = 16) vs 22.9 ± 2.8 (*n* = 182), respectively; *p* = 0.032, Student’s *t* test, data not shown).

**Table 4:** Details of postoperative pulmonary complications

PPC	asthma patients (*n* = 257)	COPD patients (*n* = 89)
Total patients with PPC	22 (8.6)	7 (7.9)
Bronchospasm	18 (7.0)	0 (0.0)
Pneumonia/atelectasis	4 (1.6)	7 (7.9)
Respiratory failure^a^	2^b^ (0.8)	1^b^ (1.1)

Data are presented as n (%)

^a^All patients complicated with respiratory failure had pneumonia and/or atelectasis

^b^One patient in each group required mechanical ventilation

**Table 5:** Clinical characteristics of asthma patients with PPCs (+) or (−)

Variables	PPC (+) *n* = 22	PPC (−) *n* = 235	*p*-value
gender, male/female	10 / 12	101 / 134	0.82*
age, years	56.5 ± 15.4	53.5 ± 16.1	0.43^†^
BMI, kg/m^2^	23.1 ± 3.0	23.3 ± 4.7	0.95^†^
Smoking Index, pack-years	18.3 ± 23.8	8.6 ± 15.9	0.15^†^
%VC, %	107.8 ± 22.1	110.7 ± 17.8	0.61^†^
%FEV_1_, %	96.5 ± 22.8	91.3 ± 19.3	0.15^†^
Peripheral blood eosinophil, /mm^3^	245 ± 183	337 ± 271	0.085^†^
GINA STEP ≥ 3, y/n	16 / 6	116 / 119	0.061*
Perioperative CS (+), y/n	17 / 5	116 / 119	0.022*
ASA-PS, 1/2/3-	1 / 19 / 2	13 / 201 / 19	0.97*
Upper abdominal surgery, y/n	4 / 18	15 / 220	0.11^‡^
Laparoscopic surgery, y/n	4 / 18	32 / 203	0.52^‡^
Operation time, min	200 ± 150	141 ± 86	0.023^†^

Data are presented as n or mean ± SD, unless otherwise stated

*CS* systemic corticosteroids

*ASA-PS* American Society of Anesthesiologists physical status

*chi-square test, ^†^Mann-Whitney *U* test, ^‡^Fisher’s exact test

**Table 6:** Clinical characteristics of COPD patients with PPCs (+) or (−)

Variables	PPC (+) *n* = 7	PPC (−) *n* = 82	*p*-value
gender, male / female	7 / 0	68 / 14	0.59^‡^
age, years	77.3 ± 7.9	70.0 ± 8.1	0.034^†^
BMI, kg/m^2^	23.8 ± 2.6	23.0 ± 3.0	0.66^†^
Smoking Index, pack-years	40.7 ± 8.6	48.2 ± 34.7	0.88^†^
%VC, %	104.8 ± 15.5	104.0 ± 21.0	0.99^†^
%FEV_1,_ %	89.5 ± 11.0	84.1 ± 21.7	0.34^†^
GOLD stage (1/2/3/4)	6/1/0/0	41/34/6/1	0.39*
Peripheral blood eosinophil, /mm^3^	210 ± 97	187 ± 135	0.38^†^
COPD treatment, y/n	3 / 4	63 / 19	0.07^‡^
ASA-PS, 1/2/3-	1 / 5 / 1	7 / 62 / 13	0.88*
Upper abdominal surgery, y/n	2 / 5	5 / 75	0.14^‡^
Laparoscopic surgery, y/n	1 / 6	15 / 67	>0.99^‡^
Operation time, min	393 ± 149	196 ± 131	0.002^†^

Data are presented as n or mean ± SD, unless otherwise stated

*chi-square test, ^†^Mann-Whitney *U* test, ^‡^Fisher’s exact test

*ASA-PS* American Society of Anesthesiologists physical status

Of the 89 patients with COPD, seven (7.9%) had PPCs, mainly pneumonia accompanied by respiratory failure. The hospitalization period was extended in these patients, but all of them survived. Nineteen of the patients underwent surgery with local anesthesia, including three in whom general was changed to local anesthesia. Of these 19 patients, no one had a PPC, but there was no difference in the prevalence of PPCs between patients treated with general and local anesthesia (*p* = 0.39, Fisher’s exact test). There was no significant difference in the prevalence of PPCs between patients who underwent laparoscopic surgery and those who did not (*p* = 0.94, chi-square test).

### Risk factors

We performed a univariate analysis to assess PPC risk factors in asthma (Table 5) and COPD (Table 6) patients. Regarding procedure-related risk factors in the univariate analysis, a longer operation time was a risk factor for PPCs. However, laparoscopic surgery did not decrease the prevalence of PPCs in the present study. We then evaluated PPC risk factors in a multivariate logistic regression analysis using the extracted variables shown in Table 7. Accordingly, the following were PPC risk factors in asthma patients: smoking history ≥20 pack-years [odds ratio (OR) 5.4, 95% confidence interval (CI) (1.4–20.8), *p* = 0.014], peripheral blood eosinophil count ≥200/mm^3^ [0.31, (0.11–0.84), *p* = 0.021], GINA STEP ≥3 [3.2, (1.04–9.9), *p* = 0.043]. However, there was no significant difference between former and current smokers in the incidence of PPCs in the univariate analysis (*p* = 0.28, Fisher’s exact test, data not shown).

**Table 7:** Risk factors of PPCs in asthma and COPD patients

	Variables	OR(95%CI)	*p*-value
asthma	Smoking index ≥20 pack-years	5.4 (1.4–20.8)	0.014
	Eosinophil ≥200/mm^3^	0.31 (0.11–0.84)	0.021
	GINA STEP ≥3	3.2 (1.04–9.9)	0.043
COPD	age, years	1.18 (1.00–1.40)	0.046
	COPD treatments, yes	0.09 (0.01–0.81)	0.032
	Upper abdominal surgery, yes	21.2 (1.3–349)	0.033
	Operation time ≥ 5 h	9.5 (1.2–77.4)	0.035

These data were analyzed by multivariate logistic regression test

The following PPC risk factors associated with COPD were identified in the multivariate logistic regression analysis: age [1.18, (1.00–1.40), *p* = 0.046], the introduction of COPD treatment [0.09, (0.01–0.81), *p* = 0.032], upper abdominal surgery [21.2, (1.3–349), *p* = 0.033], and an operation time ≥ 5 h [9.5, (1.2–77.4), *p* = 0.035] (Table 7).

## Discussion

In the present study, we show that treating asthma with perioperative systemic corticosteroids seemed to significantly increase the risk of PPCs in the univariate analysis. However, no significant risk of PPCs was detected for systemic corticosteroids in the multivariate logistic analysis. Does treatment with systemic corticosteroids increase the risk of PPCs? Previous studies reported that the incidence of PPCs in untreated patients with asthma was 24% [11], whereas it was 4.5% in patients with asthma who received perioperative systemic corticosteroids [12]. In the present study, the incidence of bronchospasm in patients with treated asthma was 7.0%, similar to the results observed in later studies. As described in the methods and the results section, all of the patients with asthma were treated with ICS, and some with more severe and unstable asthma were also treated with systemic corticosteroids. The patients with severe/uncontrolled asthma were more likely to experience PPC events. Accordingly, treatment with systemic corticosteroids behaved like a risk factor in the univariate analysis. However, in the multivariate analysis of factors associated with disease severity, this potential disadvantage was cancelled out. The results of our study suggest that increasing the ICS dose is sufficient to prevent bronchospasm in patients with mild and stable asthma. Because most of bronchospasms that present as PPCs are mild, we propose that in clinical practice, systemic corticosteroids effectively minimize the severity of PPCs in patients with severe asthma.

According to the multivariate logistic analysis of patients with asthma, peripheral blood eosinophilia and a history of heavy smoking were significantly associated with PPCs. Both of these variables are implicated in steroid responsiveness. Interestingly, the result of the present study demonstrated that a peripheral blood eosinophil count ≥200/mm^3^ decreased the risk of PPCs to one-third. There are several phenotypes of bronchial asthma [13]. The eosinophilic asthma phenotype that presents with a higher eosinophil count in the blood and sputum responded better to inhaled or systemic corticosteroid therapy [14–16]. Hence, perioperative intervention could effectively reduce PPC risk in eosinophilic asthma. We also found that a blood eosinophil count of approximately 200/mm^3^ was correlated with both sputum eosinophil count and the efficacy of ICS [14, 16, 17]. Our result is compatible with those reported in other studies.

A smoking history of ≥20 pack-years was associated a five-fold increased risk of PPCs. However, Chaudhuri et al. [18] demonstrated that the efficacy of treatment with oral corticosteroids was impaired even in former smokers with asthma. Çolak et al. [19] also found that in patients with asthma, the risk of exacerbations and pneumonia was higher in current/former smokers vs never smokers, perhaps because the efficacy of inhaled corticosteroids is reduced by smoking. The reason for this phenomenon could be that cigarette smoking inactivated histone deacetylase activity, which is important for the corticosteroid-mediated repression of anti-inflammatory gene expression [20]. Therefore, smoking could be an important PPC risk factor, even in patients with asthma who receive appropriate treatments. Again, eosinophilia and a history of heavy smoking are associated with sensitivity and resistance, respectively, to corticosteroids, and both are therefore related to steroid responsiveness in a broad sense. Hence, in the present study, steroid responsiveness was viewed as a crucial factor that influenced PPC risks.

Patients with ACO were included in the asthma group in the present study. There were three reasons for this. First, no standardized definition has yet been established for ACO. Recently, a new definition, including a post-bronchodilator FEV_1_/FVC < 0.7 and a peripheral blood eosinophil count >400/mm^3^, was proposed [21] that requires verification. Second, patients with ACO are more responsive to corticosteroids than are patients with COPD alone [22]. Because inhaled and systemic corticosteroids are required to treat bronchospasm and wheezing, symptoms that are caused by eosinophilic airway inflammation, we emphasized this fact. The latest GINA document recommends starting treatment for asthma in affected patients [23]. Third, asthma patients with a smoking history and irreversible air flow obstruction, including patients with ACO, were treated with ICS/LABA therapy to prevent bronchospasm, which was the most considerable complication of asthma. We therefore included the patients with ACO in the asthma group in our analysis of risk factors for PPC. It will be important to perform comparisons among these three types of obstructive pulmonary disease (bronchial asthma, ACO and COPD) in the future after a consensus is reached regarding the definition of ACO.

Next, regarding patients with COPD, previous studies have found that aging, upper abdominal surgery and operation time are risk factors for PPCs [1, 8]. Canet et al. [24] found that an operation time longer than 2 h was a predictor of PPCs, and in the present study, a multivariate analysis showed that an operation time longer than 5 h was a risk factor of PPCs. Hence, the threshold at which operation time increases the risk of PPCs was longer. The reason for this difference might be that perioperative management and anesthetic techniques have progressed over the last decade. In particular, non-invasive ventilation and high-flow therapy are effective in preventing post-extubation respiratory failure and reintubation in some patients [25–29]. To determinate whether the progress made in recent years has reduce the risk of PPCs was one of the purposes of this study. Laparoscopic surgery was not found to reduce PPC risk. We found that operation times were significantly longer in patients with COPD who underwent laparoscopic surgery than in those who underwent non-laparoscopic surgery (288 ± 139 min vs 195 ± 141 min, *p* < 0.01, Mann-Whitney *U* test, data not shown). The merit of using laparoscopic surgery to reduce the risk of PPC was masked by its intrinsically longer operation time. In addition to these risk factors, in the present study, we provide the newly evidence demonstrating that introducing treatment for COPD, including LAMA and LABA, reduced the risk of PPC. These inhaled drugs were recently developed and are much more effective in preventing COPD exacerbation. The mechanisms underlying their actions involve their ability to decrease hyperinflation and mechanical stress, to modulate mucus production and mucociliary clearance and to improve symptoms [30]. Low pulmonary function was not identified as a risk factor in this study. We changed the anesthetic techniques from general to local or cancelled the surgery in patients with advanced low pulmonary function. These adjustments reduced the risk of PPC in patients with low pulmonary function, but the difference was not significant.

### Limitations

The present study has some limitations. First, this study is a retrospective case-control study performed at a single institution. Because early reports have demonstrated that systemic corticosteroid is effective as a precaution against PPC in patients with asthma, we believe that it may not be ethical to perform a prospective study using a control group consisting of untreated patients. The number of patients with asthma or COPD analyzed in the present study may depend on the proportion of surgical cases per department. Otolaryngology patients comprised approximately 20% of all patients treated with general anesthesia, and these patients often had asthma. However, the number of cases in which cardiovascular diseases, esophageal cancer or laryngopharyngeal cancer coexisted with COPD was small. Hence, multicenter cohort studies are desirable for collecting balanced data.

Second, although the total number of patients was 346, it would be preferable to register more patients. In addition, fewer PPC events in patients with COPD were observed than expected in this study. However, the reason for the small number of PPC events could be that we managed our patients in a manner aimed at reducing PPCs. Third, we excluded patients who underwent lung resection, because it is a direct risk factor for pulmonary complications. In the future, these concerns should be addressed in separate studies.

## Conclusions

In summary, our results demonstrate that asthma patients with a history of smoking or severe asthma had an increased risk of PPCs. Sufficiently and aggressively increasing ICS can reduce the risk of PPCs in asthma patients with eosinophilia. Although aging, upper abdominal surgery and long operation times are risk factors for pneumonia and respiratory failure in COPD patients, introducing treatment for COPD significantly reduces the risk of PPC by improving pulmonary function.

## Abbreviations

ACO: Asthma-COPD overlap
ACT: Asthma Control Test
ASA-PS: American Society of Anesthesiologists physical status
CI: Confidence interval
COPD: Chronic obstructive pulmonary disease
FEV_1_: Forced expiratory volume in one second
FVC: Forced volume capacity
GINA: Global Initiative for Asthma
GOLD: Global initiative for chronic Obstructive Lung Diseases
ICS: Inhaled corticosteroids
LABA: Long-acting beta 2 agonist
LAMA: Long-acting muscarinic antagonist
OR: Odds ratio
PPC: Postoperative pulmonary complication
SD: Standard deviation
VC: Volume capacity
